# PDDA/Honey Antibacterial Nanofiber Composites for Diabetic Wound-Healing: Preparation, Characterization, and In Vivo Studies

**DOI:** 10.3390/gels9030173

**Published:** 2023-02-22

**Authors:** Mazeyar Parvinzadeh Gashti, Seyed Ahmad Dehdast, Ali Berenjian, Mohammad Shabani, Ehsan Zarinabadi, Ghazaleh Chiari Fard

**Affiliations:** 1 GTI Chemical Solutions, Inc., Wellford, SC 29385, USA; 2 InsectaPel, LLC, Wellford, SC 29585, USA; 3 Department of Biochemistry, School of Medicine, Iran University of Medical Sciences, 14496-14535 Tehran, Iran; 4 “Pajoohesh BAMA” Knowledge Enterprise Co., 15847-15414 Tehran, Iran; 5 Department Textile, Islamic Azad University, South Tehran Branch, 11365-4435 Tehran, Iran; 6 Clothing and Fabric Design Department, Art Faculty, Imam Javad University College, 89158-73764 Yazd, Iran

**Keywords:** PDDA/honey nanofiber composites, antibacterial characteristics, water absorption, wound-healing properties, controlled release

## Abstract

In this paper, Poly (diallyldimethylammonium chloride) (PDDA)/honey nanofiber wound dressing composites were prepared and their effects on the diabetic wound-healing was evaluated using in vivo experiments. The release of effective compounds and the solubility of nanofibers were controlled through the crosslinking process by glutaraldehyde. The crosslinked nanofibers (crosslinking time was 3 h) showed an absorption capacity at a maximum value of 989.54%. Interestingly, the resultant composites were able to prevent 99.9% of *Staphylococcus aureus* and *Escherichia coli* bacteria. Furthermore, effective compounds were continuously released from nanofibers for up to 125 h. In vivo evaluation indicated that the use of PDDA/honey (40/60) significantly enhanced wound-healing. On the day 14th, the average healing rate for samples covered by conventional gauze bandage, PDDA, PDDA/honey (50/50), and PDDA/honey (40/60) were 46.8 ± 0.2, 59.4 ± 0.1, 81.7 ± 0.3, and 94.3 ± 0.2, respectively. The prepared nanofibers accelerated the wound-healing process and reduced the acute and chronic inflammation. Hence, our PDDA/honey wound dressing composites open up new future treatment options for diabetic wound diseases.

## 1. Introduction

Diabetes mellitus is one of the most prevalent chronic diseases in the world, which is a consequence of the existence of a malfunction in coagulation, hemostasis, inflammation, proliferation, and the remodeling of wound-healing process [1,2]. Chronic wounds can usually be extended for three main reasons: inflammation, poor perfusion, and the presence of necrotic tissue. On the other hand, wound infection is a major complication in diabetic patients [3]. Micro-organisms can be extensively spread in chronic wounds due to the relative moisture, warmth, and available nutrients. Many studies have been performed on the use of synthetic drug-incorporated nanofibers for treating the diabetic ulcer infections [4]. However, the use of synthetic drugs is not favorable due to their side effects and relatively high costs. As an alternative choice, traditional and herbal medicines have recently gained popularity due to their availability, moderate efficacy, no or fewer side effects, and low costs [5].

In recent years, nanoparticles with different morphologies have extensively been studied owing to their advantages in catalysis reactions, energy storage applications, nanoreactors, biosensors, drug delivery systems, and wound-healing in biomedical industries. It has been proven that nanofibers, nanotubes, and nanospheres exhibit exceptional optical, thermal, electrical, magnetic, and mechanical characteristics in regenerative medicine [6,7]. One-dimensional structures are used in many fields due to their high specific surface area. It has been claimed that electrospun nanofiber platforms have very small pores and can prevent the bacterial penetration [8]. Additionally, the nanofibrous structures resemble the extracellular matrix of the skin, which will lead to an improvement in the healing process. The electrospinning technique is a simple, convenient, low cost, and versatile method for producing one-dimensional nanostructure materials [9,10,11,12]. Moreover, the process parameters can be tailored to produce nanofibers with different diameters, morphologies, and area densities.

Across the world, several research studies on the biocompatible and antimicrobial polymers have been carried out due to their biomedical importance in the treatment of infectious diseases [13,14]. Poly (diallyldimethylammonium chloride) (PDDA) is a cationic antimicrobial polymer with remarkable applications in water, paper, mining, and petrochemical as a coagulant, dehazer, or demulsifier reagent [15]. Additionally, it is has become known as a model polymer in recent research on polyelectrolytes, functional nanomaterials, biosensors, fuel cells, capturing cancer cells, and wound dressings [16,17,18,19,20]. In this context, the functionalized graphene nanosheets were synthesized with PDDA and further combined with room temperature-ionic liquids [21]. Recently, PDDA and sodium silicate multilayers had been applied to silica spheres to generate a superhydrophobic layer [22]. PDDA holds the quaternary ammonium moieties as pendant groups in the chemical structure, which displays outstanding antimicrobial activity [23]. In this regard, it could be a good candidate for the production of wound dressings. Sanches et al. stated that cationic bilayer fragment/carboxymethylcellulose (CMC)/PDDA nanoparticles have strong antibacterial properties [24]. Zhou et al. found that PDDA improved the adsorptive capacity of Ag/AgCl/reduced graphene oxide, which in turn enhanced the bactericidal activity of resultant composite particles [25]. Sundarrajan and Ramakrishna optimized the electrospinning of PDDA/poly vinyl alcohol (PVA) nanofibers for tissue engineering and controlled drug delivery [26].

Honey is a natural food substance with several monosaccharides, including glucose, sucrose, and fructose. Honey has several potential applications in wound care due to unique antibacterial properties [27]. We should emphasize that honey is not only able to reduce inflammation, can avert the need for surgical debridement, and neutralize bad smells, it can also accelerate tissue growth and wound-healing [28]. In addition, the fabrication of electrospun honey-based nanofibrous matrices has recently been demonstrated. In a research work, polyethylene terephthalate (PET)/honey/chitosan nanofibers were produced for wound dressing purposes [29]. Additionally, homogenous PVA/honey electrospun nanofibers were recently fabricated [27]. Sarhan et al. postulated the antimicrobial and wound-healing activity of honey/chitosan nanofibers after enrichment with allium sativum and cleome droserifolia [30]. The majority of previous research has been focused on the combination of honey with different biopolymers and no study has been presented which is concerned with the fabrication of PDDA and PDDA/honey nanofiber composites for wound-healing investigation [31]. In this paper, PDDA/honey nanofibers produced using the electrospinning method and were characterized using different analytical techniques. Furthermore, fabricated nanofibers were chemically modified with glutaraldehyde to reduce the solubility and increase the water absorption properties. All in all, we analyzed the resultant nanofiber composites for diabetic wound dressings.

## 2. Results and Discussion

### 2.1. Microscopic Characterization 

**Figure 1:**
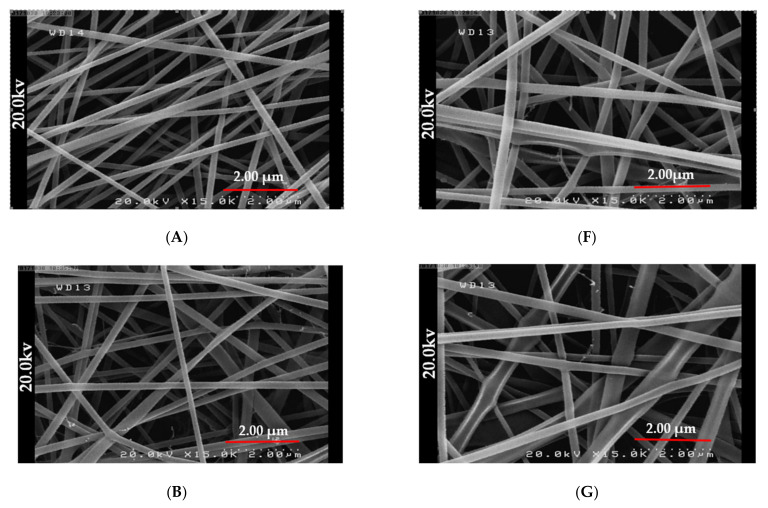

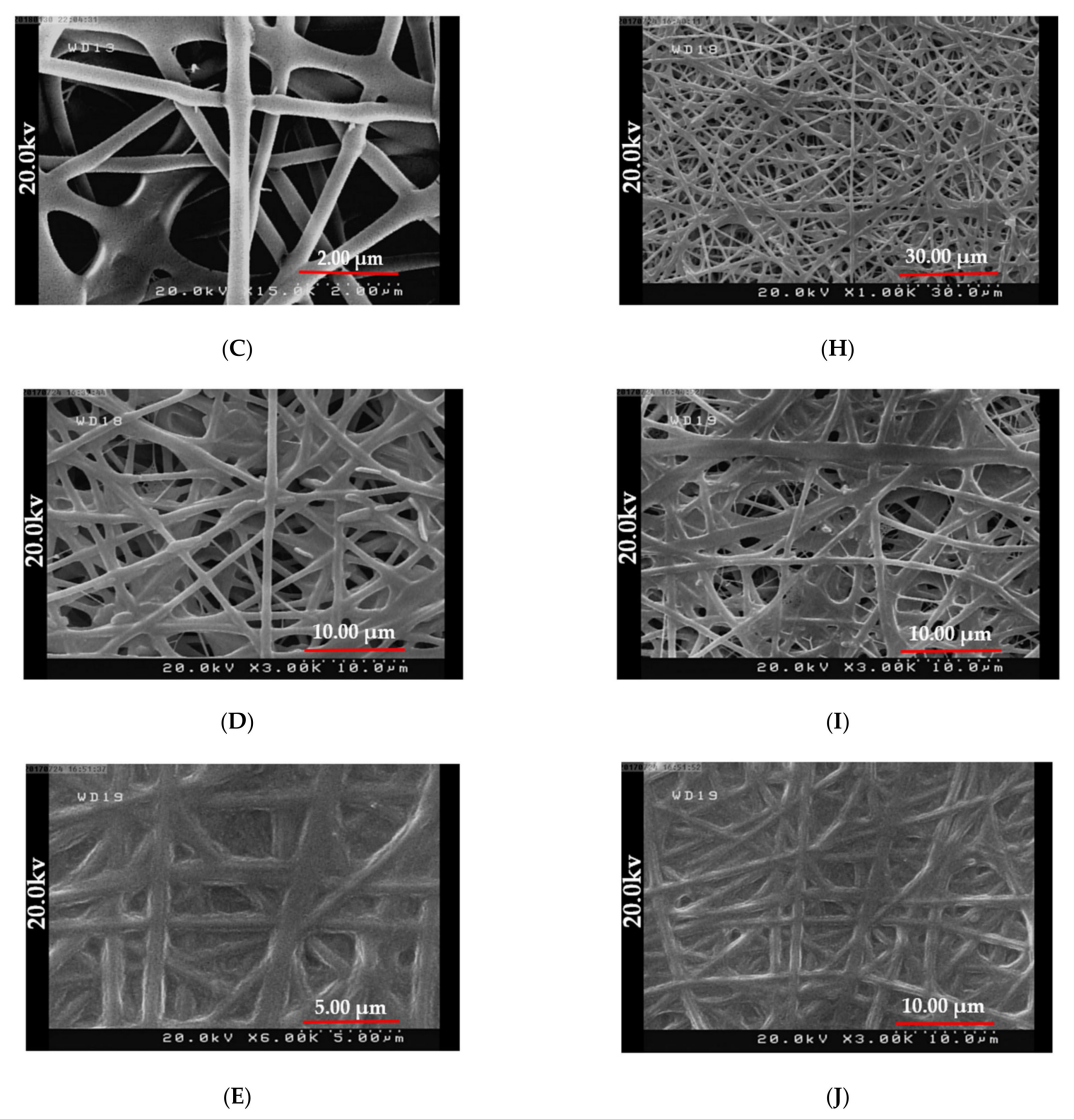
The FESEM images (**A**) PDDA/honey nanofiber composite 50/50 (20 KV), (**B**) crosslinked PDDA/honey nanofiber composite (50/50) for 2 h, (**C**) crosslinked PDDA/honey nanofiber composite (50/50) for 3 h, (**D**) crosslinked PDDA/honey nanofiber composite (50/50) for 4 h, (**E**) crosslinked PDDA/honey nanofiber composite (50/50) for 5 h, (**F**) PDDA/honey nanofiber composite 40/60 (17 KV), (**G**) crosslinked PDDA/honey nanofiber composite (40/60) for 2 h, (**H**) crosslinked PDDA/honey nanofiber composite (40/60) for 3 h, (**I**) crosslinked PDDA/honey nanofiber composite (40/60) for 4 h, and (**J**) crosslinked PDDA/honey nanofiber composite (40/60) for 5 h.

In our previous research, we optimized the fabrication of PDDA and PDDA/honey nanofiber composites with similar molecular weights and properties [31]. In this study, in order to investigate the effect of honey on the healing properties of PDDA/honey composite nanofibers for diabetic wounds, two ratios were produced: (50/50) and (40/60) (after the confirmation of optimal values by Design Expert Software V 11.0.3). Figure 1A,F showed the FESEM images of PDDA/honey nanofiber composites with the concentration ratio of 50/50 and 40/60% *w*/*w* at 20 and 17 applied voltages, respectively. As can be seen, nanofibers had a smooth surface and a uniform morphology. They had the average diameter of 91 and 93.5 nm for PDDA/honey (50/50) and (40/60) nanofiber composites, respectively.

Figure 1B–E,G–J illustrated the FESEM images of PDDA/honey (50/50) and (40/60) [31] nanofiber composites after crosslinking for different time periods, including 2, 3, 4, and 5 h. It was clear that the nanofibers were conglutinated to each other, especially at their touching points, after crosslinking for 2 h. This could be due to the fact that a thin layer of the crosslinking agent was able to cover the nanofiber’s surface, which affected their hydrophilic nature. When the crosslinking duration was increased from 2 to 5 h, the morphology of nanofibers changed from a fibrillary form to a thin film. Furthermore, the visible voids within the nanofiber matrix were filled by glutaraldehyde [31]. Similar to our work, Hixon et al. observed that the addition of Manuka honey in cryogel-hydrogel electrospun nanofibers resulted in thicker tissue with fewer obvious pores than in the untreated samples [32].

### 2.2. FTIR Analysis

**Figure 2:**
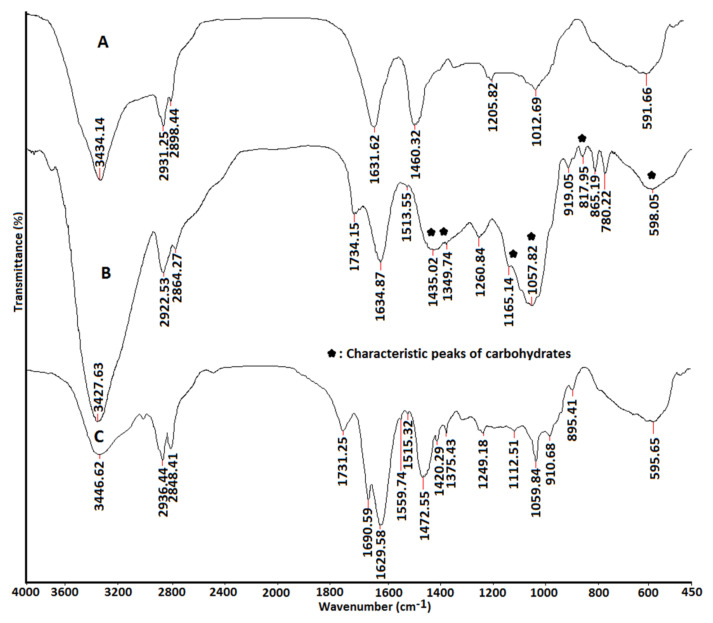
The FTIR spectra of (**A**) PDDA, (**B**) PDDA/honey (40/60% *w*/*w*), and (**C**) crosslinked PDDA/honey (40/60% *w*/*w*) nanofiber composites.

The FTIR spectra of PDDA and non-crosslinked and crosslinked PDDA/honey (40/60) nanofiber composite samples are shown in Figure 2. For PDDA nanofibers, the bands at 3434 and 1631 cm^−1^ were attributed to the stretching and bending vibrations of the absorbed water molecules, respectively. Additionally, the band that appeared at 1205 cm^−1^ was related to the stretching vibration of C-N bond [33]. The peak at 1460 cm^−1^ was due to the CH_2_ bending vibrations. The bands at 2931 and 2898 cm^−1^ was assigned to the methylene C-H asymmetric and symmetric stretching vibrations, respectively. Honey was mainly composed of hydrated glucose, fructose, and carbohydrates. According to Figure 2B, the skeletal vibrations of carbohydrates appeared in the range of 600–1500 cm^−1^ [29]. Additionally, the band at 3443 cm^−1^ was related to the stretching vibration of the OH group of carbohydrates [34]. The bands that appeared at 2994 and 2841 cm^−1^ were assigned to the stretching vibrations of CH bonds. The band at 1513 cm^−1^ is attributed to the vibration of C=C bond in the aromatic ring of polyphenols in honey [35]. Amino acids that exist in honey generally contain amide groups with several characteristic bands, such as amide A (about 3500 cm^−1^), amide B (about 3100 cm^−1^), amide I (1600–1700 cm^−1^), amide II (1550 cm^−1^), and amide III (1300–1350 cm^−1^), depending on the type and number of amino acids. The characteristic peaks of PDDA overlapped with characteristic peaks from honey. By comparing the curves B and C, it was found that the intensity of bands at 1059, 2936, 2848, and 1731 cm^−1^ increased, which were all related to the acetal band [36]. This change was resulted from the reaction of glutaraldehyde with the hydroxyl containing compounds, the stretching vibrations of CH bonds, and the stretching vibration of C=O groups in honey [37]. Additionally, the intensity of the band at 3446 cm^−1^ was decreased due to the reaction of the crosslinking agent with OH containing compounds in honey. A new absorption peak at 1690 cm^−1^ was related to the imine group [38], resulting from the reaction between glutaraldehyde and amine groups of different compounds in honey. Naemi et al. recently observed that addition of honey to a Chitosan/PVA nanocomposite resulted in hydrogen bonds between functional groups of honey with hydroxyl and amine groups from PVA and chitosan [39]. Similarly, Ghorbani et al. observed a shift in the absorption peaks at 1645 and 1522  cm^−1^ to a high wavenumber after loading honey in ethylcellulose/gum tragacanth nanofibers [40].

### 2.3. Fluid Absorption and Solubility of Nanofibers

It is established that the wound moist is an ideal environment for the bacterial growth. Additionally, bacterial infections can result in impaired healing. In general, antibiotics are largely used for treating wound infections. However, this is not the best treatment methods for burns and chronic wounds. In this regard, using dressing technologies with different biocompatible materials can be among alternative treatments. In comparison with different compounds used for wound-healing, honey-incorporated scaffolds are highly recommended due to their antimicrobial and controlled wound hydration properties [41].

**Figure 3:**
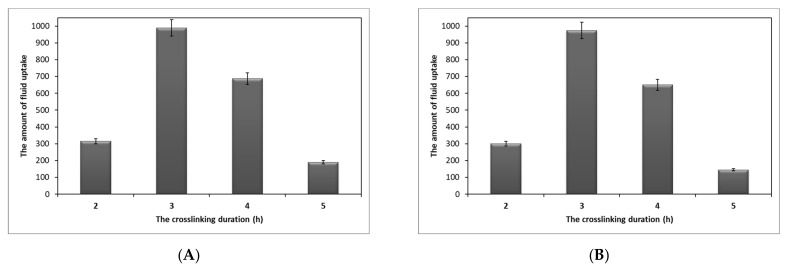
The amount of fluid uptake for PDDA/honey nanofiber composites at different crosslinking duration: (**A**) crosslinked PDDA/honey nanofiber composite (40/60) and (**B**) crosslinked PDDA/honey nanofiber composite (50/50).

The amounts of fluid uptake of PDDA/honey (40/60) nanofiber composites with the crosslinking durations of 2, 3, 4 and 5 h were 314.55, 989.54, 687.29 and 190.43%, respectively (Figure 3A). In other words, longer crosslinking duration was able to further change the morphology of nanofibers. Additionally, we noted that crosslinked nanofibers for 4 and 5 h exhibited stiffer tissues with low flexibility, which is a disadvantage for wound dressing applications. In addition, longer crosslinking durations resulted in drastic changes in the hydrophilic nature of the nanofibers, thus hindering the diffusion of water molecules into the nano-fibrous mats. We also considered the solubility of nanofibers as another factor to evaluate the performance of crosslinking in wound dressings. Results revealed that the sample crosslinked for 2 h exhibited a 39.3% weight loss, whereas there was no weight reduction for the other samples crosslinked for longer durations. Furthermore, the solubility of nanofiber composite prepared from PDDA/honey ratio of 50/50 and crosslinked for different durations was investigated. The nanofiber composite crosslinked for 2 h, showing a 38.58% weight loss, while no weight reduction was observed for longer crosslinking durations. The fluid absorption for the composite samples crosslinked for 2, 3, 4, and 5 h was 300.41, 975.28, 651.11, and 145.43%, respectively (Figure 3B). We found no weight loss for the PDDA nanofiber sample crosslinked for 3 h. Additionally, the fluid absorption value for this sample was 665.72%. A recent study by Tang et al. expressed the effect of honey on the water absorption capability of honey-loaded alginate/PVA nanofibrous membranes. According to their results, dissolving honey in the nanofiber structure led to the degradation and destruction of the internal structures of membranes, thus decreasing their water absorption ability [42].

### 2.4. Release Behavior of Nanofibers

**Figure 4:**
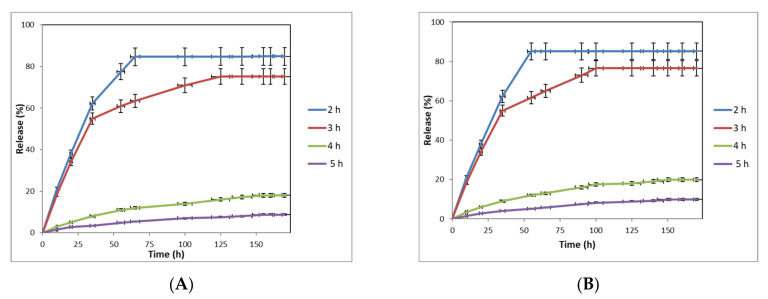
The release behavior of (**A**) PDDA/honey nanofiber composite (40/60) and (**B**) PDDA/honey nanofiber composite (50/50).

The release behavior of nanofiber samples produced at different crosslinking duration was investigated, and the results are shown in Figure 4A. When increasing the crosslinking duration, the total amount of released compounds from PDDA/honey nanofiber composite (40/60) crosslinked for 2 h was 84.8%. However, it decreased to 75.14, 18.06, and 8.72% for those composites crosslinked for 3, 4, and 5 h, respectively. This drastic decrease in the released materials for 4 and 5 h crosslinked samples could be related to the change of morphology of samples and their hydrophobic properties. Our results are also consistent with solubility test measurement of nanofiber composites. The results further showed that the increase of crosslinking duration from 3 to 5 h led to an increase in releasing time from 125 to 150 h. According to Figure 4B, a major increase in the released materials occurred in the first 35 h period, which could be related to the high amount of the entrapped compounds on the surface of nanofibers. The incorporation of effective compounds on the nanofiber surface is attributed to the limited physical interactions between the effective compounds and the polymer matrix in the electrospinning process [43]. After 35 h, the rate of release decreased and an equilibrium point was finally achieved. In this regard, the number of released compounds for the samples crosslinked for 2, 3, 4, and 5 h were 22.5, 20.17, 11.05, 3.74, and 2.04% of the total values. Additionally, the equilibrium values were 65, 125, 155, and 155 h, respectively. Similar behavior was observed for the PDDA/honey nanofiber composite (50/50). For this sample, the equilibrium values were 55, 115, 150, and 150 h after crosslinking for 2, 3, 4, and 5 h, respectively. Additionally, the total amounts of the released compounds from these samples were 85.1, 76.51, 19.88 and 9.91%, respectively. As can be seen, the total amount of released compounds for the PDDA/honey nanofiber composite (50/50) was slightly higher than PDDA/honey nanofiber composite sample (40/60). This could possibly be related to the average nanofiber diameter (AND) of samples. The AND of 50/50 and 40/60 PDDA/honey nanofiber composites were 91 and 93.5 nm, respectively. We concluded from the results of fluid absorption, solubility, and release behavior of nanofiber composites that the crosslinking duration of 3 h provided the optimum results.

### 2.5. Antibacterial Activity of Nanofibers

**Figure 5:**
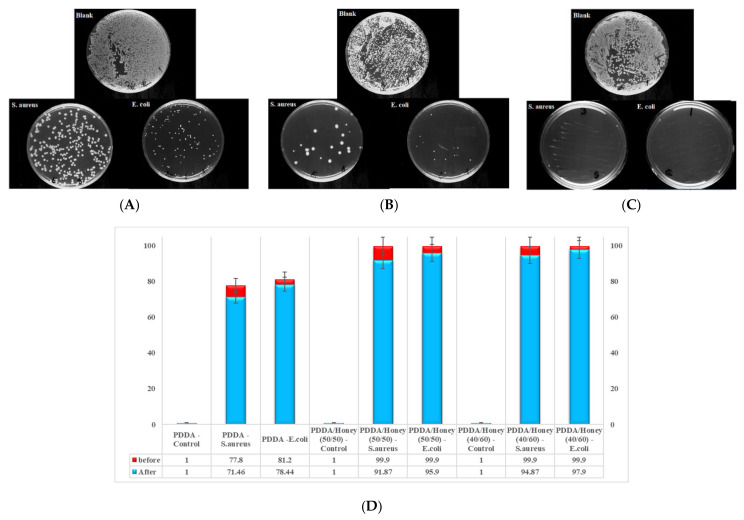
The antibacterial activity of fabricated materials against *E. coli* and *S. aureus*. (**A**) PDDA, (**B**) PDDA/honey (50/50) nanofiber composite, (**C**) PDDA/honey (40/60) nanofiber composite, and (**D**) before dilution and after dilution.

The antibacterial activity of PDDA and PDDA/honey nanofiber composites were evaluated by using the viable cell-counting process. The effects of fabrication of resultant materials on the Gram-positive (*Staphylococcus (S.) aureus*) and Gram-negative (*Escherichia coli (E.) coli*) bacterial culture were investigated, and the results are shown in Figure 5. According to Figure 5A–C, the number of bacterial colonies decreased after treatment with honey incorporated nanofiber composites. For the PDDA/honey nanofiber samples, *E. coli* and *S. aureus* bacteria with an initial concentration of 5 × 10^5^ CFU/mL were completely eliminated. On the other hand, certain bacterial colonies were seen on the PDDA nanofiber. Additionally, the antibacterial property of PDDA sample against *E. coli* was slightly higher than *S. aureus*. We should emphasize that the antibacterial properties of the PDDA nanofiber were 77.8 and 81.2% for *S. aureus* and *E. coli* bacteria, respectively. According to previous studies, the antibacterial characteristics of the PDDA polycation polymer are determined by the permanently charged quaternary ammonium domains in the cyclic structure, which can interplay with the negatively charged bacterial surface, and as a result, bacteria membrane walls can be destroyed, resulting in cell leakage and death [22]. The antibacterial properties of honey are due to the H_2_O_2_ and antioxidant polyphenols within its composition. Polyphenols are strong pro-oxidants in the presence of transition metal ions, promoting hydroxyl radicals from H_2_O_2_ via the Fenton process [44]. The high sugar content of honey draws fluid from the wound by osmosis, resulting in bacterial death [45]. It was reported that flavonoids, including benzoic and cinnamic acids, contribute to the antibacterial activity of honey. However, the effectiveness of these components in the overall antibacterial properties of honey is low compared to the contribution from hydrogen peroxide [46]. According to the manufacturer, the total phenolic and flavonoid contents of our used honey were 59.65 and mg GAE/100 g honey, respectively. We achieved 99.9% antibacterial efficiency from PDDA/honey (40/60 and 50/50) composite nanofibers against *S. aureus* and *E. coli*. Our results demonstrated synergistic antibacterial properties in PDDA/honey composite nanofibers, in comparison with honey or the PDDA nanofiber. In addition, the antibacterial activity of fabricated materials on *E. coli* and *S. aureus* did not change after dilution (Figure 5D).

### 2.6. Wound-Healing and Histopathological Studies

**Figure 6:**
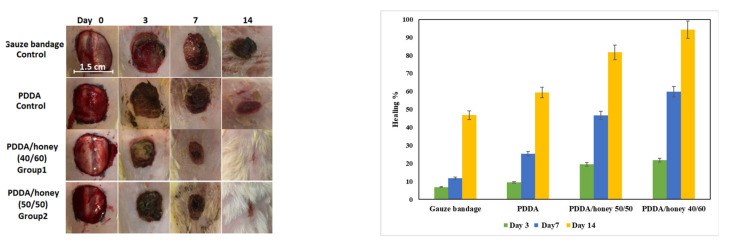
Images of generated wounds after treatment with nanofibers for different durations of 0, 3, 7, and 14 days.

We conducted wound-healing and histopathological studies on the resultant samples. The images of wounds after treatment with nanofibers for different durations, namely 0, 3, 7, and 14 days, are shown in Figure 6. As can be seen, the wound-healing percentage for diabetic rats after being treated with a gauze bandage, PPDA nanofiber along with PDDA/honey (50/50), and PDDA/honey (40/60) nanofiber composites was 6.8 ± 0.2, 9.5 ± 0.2, 19.5 ± 0.3, and 21.8 ± 0.1 after 3 days, respectively. It was clear that the rate of wound closure for the wound covered by honey incorporated nanofibers was much higher than that covered with the gauze bandage. This was due to the higher absorption ability and barrier properties of nanofibers against bacteria [8]. On the other hand, the healing percentages for the gauze bandage, PDDA nanofiber, and PDDA/honey (50/50) and PDDA/honey (40/60) nanofiber composite treated wounds were 11.8 ± 0.4, 25.3 ± 0.2, 46.7 ± 0.1, and 59.8 ± 0.1 after 7 days, respectively. Low healing properties for the gauze-covered sample were attributed to the lack of moisture on the surface of the wound. Although the diameter of wound was diminished after treatment with gauze, the improvement of the wound-healing properties of nanofiber composites could be related to the modulation of the inflammatory responses of the skin dendritic cell line. Notably, PDDA/honey (50/50) and PDDA/honey (40/60) nanofiber composites are able to influence the fast autolytic debridement and the suppression of inflammation [47]. Previous studies demonstrated that honey enhances the tissue repair process via the stimulation of the leukocytes, which in turn releases cytokines and triggers an immune response against infection [48]. Moreover, honey contains high number of antioxidants, which are important factors in wound-healing applications. Antioxidants have been correlated to the improvement of the wound-healing process by preventing the overexposure of the wound to oxidative stress. Accordingly, delays in the wound-healing process can occur [49]. It was previously revealed that honey’s antioxidants are mainly flavonoids, phenolic acids, catalase, peroxidase, carotenoids, and non-peroxidal [50]. Additionally, it was believed that the antioxidant activity of honey depended on several factors, including the floral source, season, and climatic changes. We should also mention that processing of honey may effect its antioxidant activity [51]. The total antioxidant capacity of the honey we used was 56.61 mg AAE/g honey.

The healing percentages for the wounds treated with gauze bandage, PDDA, PDDA/honey (50/50), and PDDA/honey (40/60) nanofiber composites were 46.8 ± 0.2, 59.4 ± 0.1, 81.7 ± 0.3, and 94.3 ± 0.2 after 14 days, respectively. We also found that the healing rate for the wound covered with the PDDA/honey nanofiber composite (40/60) was higher than that covered with the PDDA/honey composite (50/50). Therefore, the more honey used in the nanofiber composite, the higher the wound-healing rate. This is also related to the equilibrium point of the effective compounds after release from the nanofiber composites. As mentioned earlier, the equilibrium point of PDDA/honey nanofiber composite (50/50) was 100 h, while it was 125 h for the PDDA/honey nanofiber composite (40/60). Since the wound dressing was changed every 5 days, the possibility of bacterial growth on the wound surface was increased and the effective compounds were not in direct contact with the generated wound. Therefore, the controlled release of the effective compounds from nanofiber composites were able to promote the wound-healing. More importantly, honey aids in the healing process through the acidification of the alkaline environment in chronic ulcers. Acidification inhibits protease activity, induces fibroblast proliferation, and establishes an aerobic environment [52]. A higher healing percentage for the PDDA nanofiber-covered wound was attributed to the antibacterial activity of the PDDA polymer and the high absorption capability of nanofibers.

**Figure 7:**
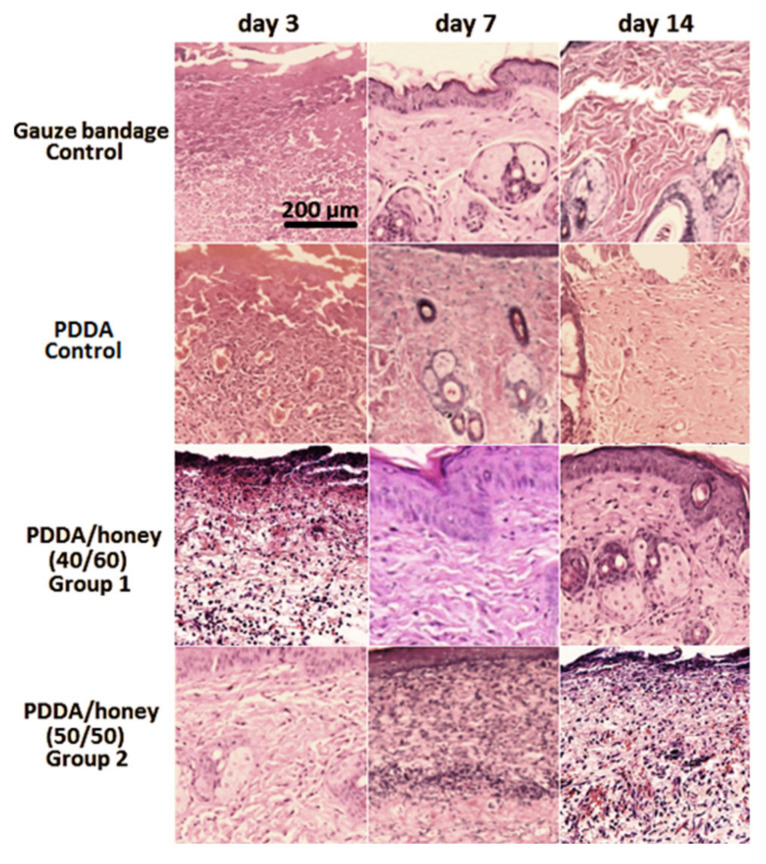
Histological images (×200) of the control wound along with the nanofiber-treated wounds at 3, 7, and 14 days.

We also illustrated the histopathological images of the control wound along with the nanofiber-treated wounds at 3, 7, and 14 days (Figure 7). For the wounds covered by nanofiber composites, a large number of inflammatory cells was observed on day 3. On the other hand, they were too low for the gauze bandage-treated wounds. In addition, a massive infiltration of inflammatory cells was observed after 7 days and the matured granulation tissue along with epidermal layer was detected after 14 days. Additionally, the number of inflammatory cells was diminished significantly after 14 days. These anti-inflammatory properties of honey were confirmed by other researchers [53]. A further decrease in the number of inflammatory cells was observed after treatment of the wound with the PDDA/honey nanofiber composite (40/60) in comparison with the PDDA/honey nanofiber composite (50/50)-treated wound. To put it simply, the formation of fibroblast cells, collagen fibers, and connective fibrils were observed for the PDDA/honey (40/60)-treated wound after 14 days. Consistent with other studies, the granulation of tissue components in the honey-incorporated nanofiber composite-treated wounds was improved, which could be due to the increased collagen turnover. For the PDDA nanofiber-treated wound, a large number of inflammatory cells was observed after 7 days. In addition, regenerative responses and tissue granulation were observed after 14 days in these samples.

### 2.7. Histomorphometric Analysis

The histomorphometric analysis was performed on nanofiber-treated injured skin after 3, 7, and 14 days, and the results are presented in Table 1. The result indicated better healing for the PDDA/honey nanofiber composite-treated wound. In addition, the re-epithelialization of this wound was higher than other nanofiber-treated wounds after 14 days. Additionally, a significant decrease in the number of inflammatory cells in the wounds covered by honey-incorporated nanofiber composite dressings was confirmed after 14 days. We should note that the PDDA/honey nanofiber composite (40/60) demonstrated the best wound-healing properties among all samples.

### 2.8. Identification of the Effect of Variables on Wound-Healing (%) Improvement

In this study, we used the analysis of three-dimensional graphs to observe the effect of variables and their effects on the experiments. Moreover, the relationship of different variables and their effects on wound-healing (%) was studied.

**Figure 8:**
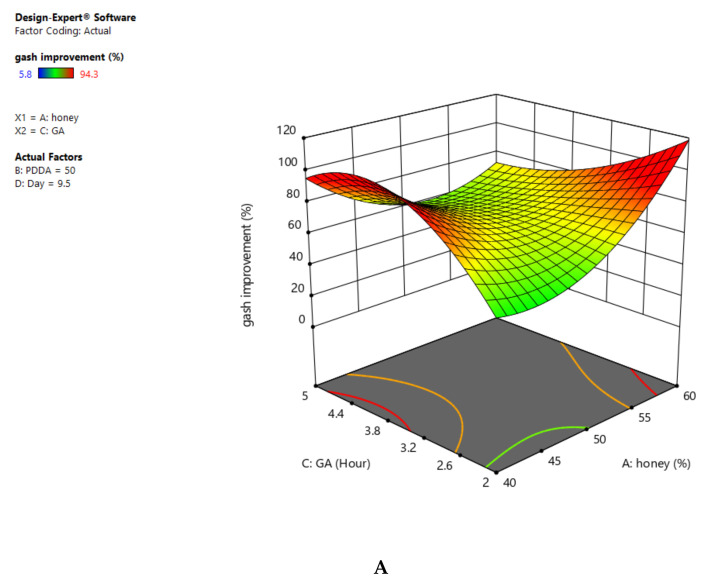

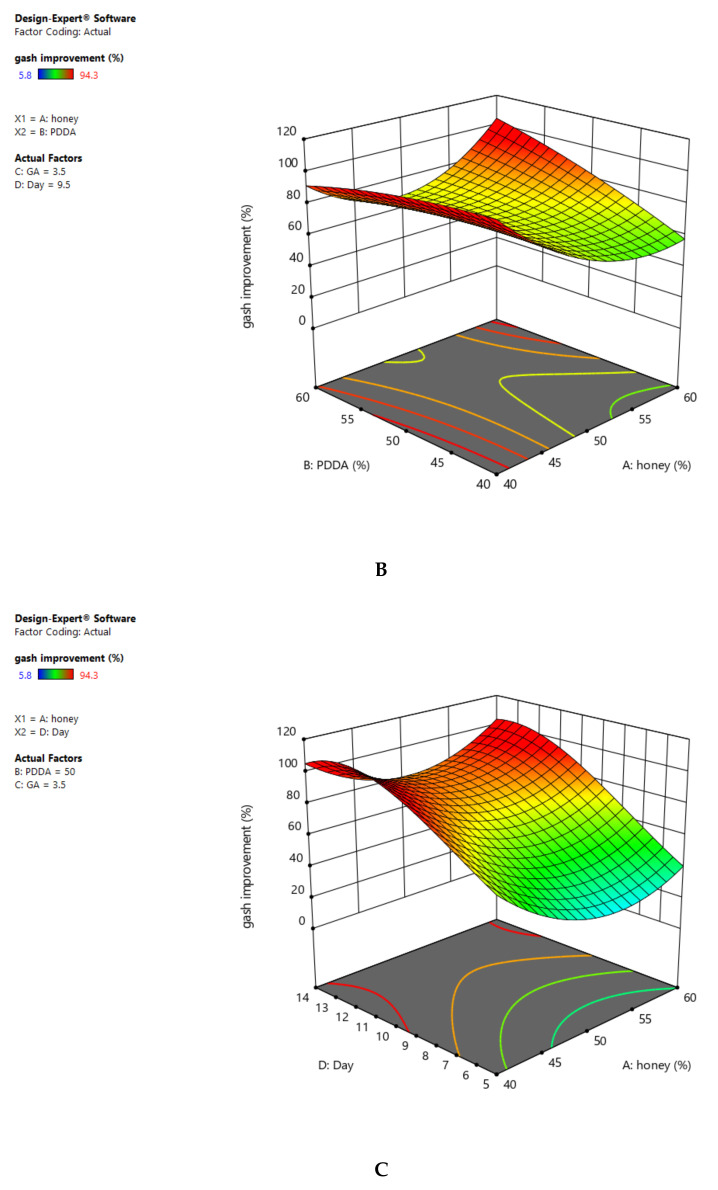

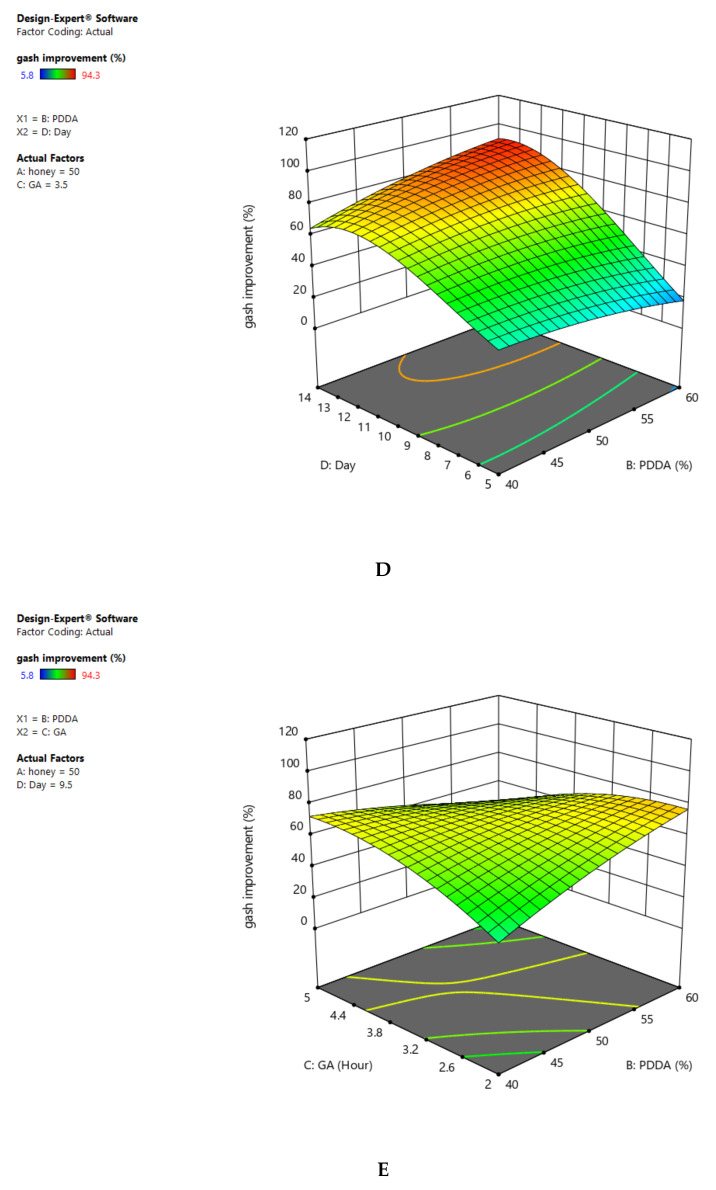

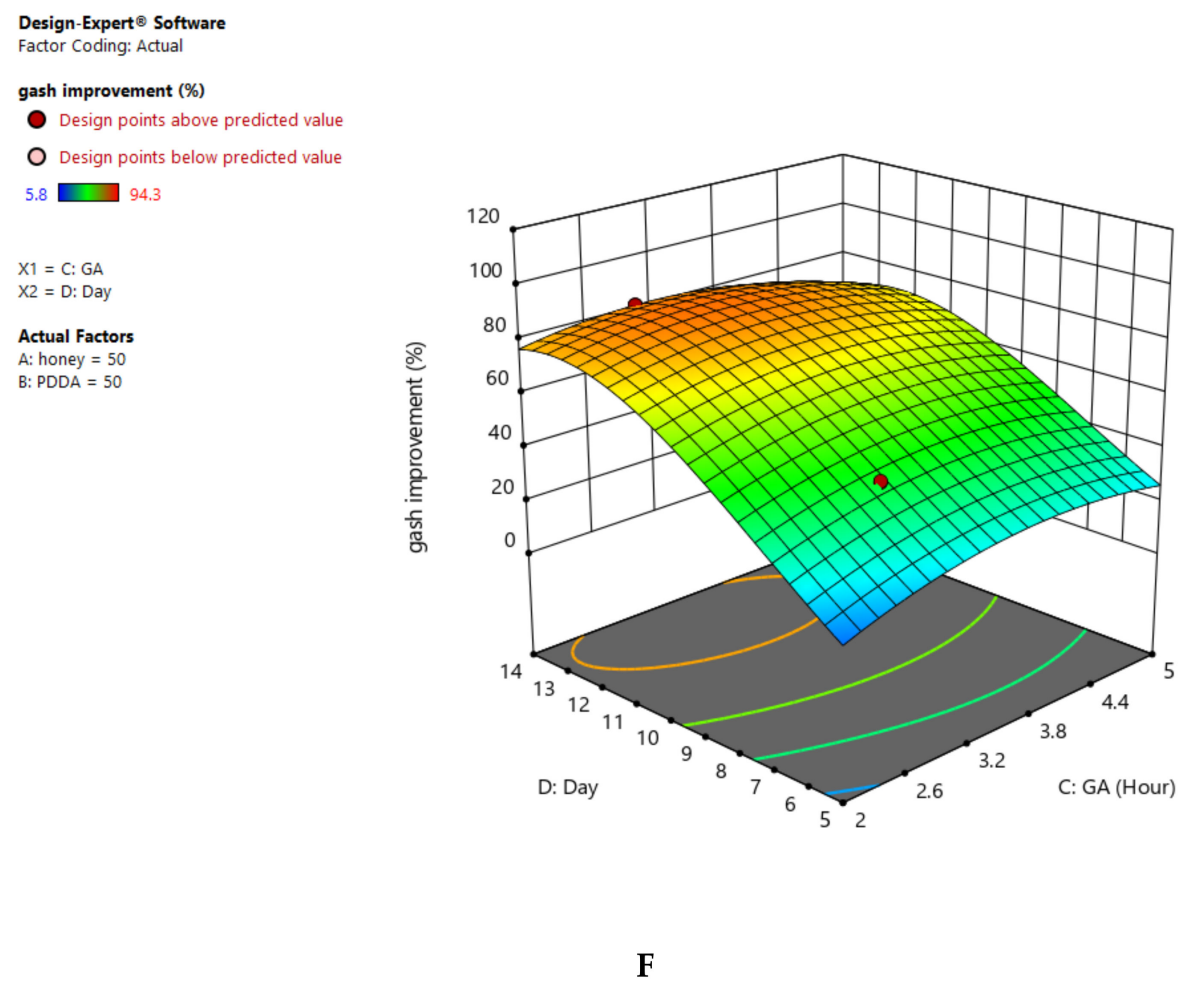
(**A**) A 3D diagram for the effects of honey (%) and PDDA (%) on wound-healing, (**B**) 3D diagram for the effects of honey (%) and crosslinking duration (h) on wound-healing, (**C**) 3D diagram for the effects of honey (%) and static time on wound-healing, (**D**) 3D diagram for the effects of crosslinking duration (h) and PDDA (%) on wound-healing, (**E**) 3D diagram of the effects of PDDA (%) and static time on wound-healing, and (**F**) 3D diagram of the effects of crosslinking duration (h) and on wound-healing.

As can be seen, the combination of the two variables of honey (%) and PDDA (%) was 100 in total. Note that we considered the changes on the chart based on this total value. After consideration of these two variables, the wound-healing rate was changed with increasing the concentration of honey (between 40 to 60%) and decreasing the concentration of PDDA from 60 to 40% (the red region in Figure 8A). 

Figure 8B depicts the effect of crosslinking duration and the percentage of honey on the wound-healing properties of resultant composites. As can be seen, these two variables had a reverse effect: the more honey (%) used in the experiment, the less crosslinking duration (h) required in the final composite. Furthermore, the green region of the chart demonstrates that the honey variable was about 40% when the crosslinking duration was between 3.2 and 4.4 h. Interestingly, the crosslinking duration reached 2.5 h in the red region. There was also a peak in the green region, confirming that honey (at 50% value) had a significant effect on wound-healing at lower crosslinking durations.

A 3D diagram of the relationship of honey (%) and static time on wound-healing properties is depicted in Figure 8C. Results showed that application of wound dressing to up to 14 days resulted in an enhancement in wound-healing. It is noteworthy that by using up to 60% honey (%), no significant improvement in wound-healing was observed between 5 to 12 days. On the other hand, wound-healing rate was highly influenced after 14 days. Therefore, it can be concluded that samples with 60% honey content display a rapid improvement in wound-healing after 13 and 14 days.

According to the presented diagram in Figure 8E, there was a decrease in wound-healing in the green region. Results revealed that the wound-healing (%) was decreased with any decrease in the amount of PDDA (%) and crosslinking duration. In addition, when we simultaneously changed these two variables (PDDA up to 55% and crosslinking duration for 4.4 h), wound-healing was improved by up to 65%. However, longer crosslinking duration had a negative effect on wound-healing. We also evaluated the effect of PDDA content (%) and the duration of static wound dressing on wound-healing (Figure 8F). We found that the wound-healing (%) was improved after treatment for 14 days.

We should express that the duration of crosslinking (2–3 h) and static wound dressing had a major effect on wound-healing (%). However, wound-healing was slightly decreased with any increase in crosslinking duration from 3 to 5 (h). We demonstrate the optimum values for wound-healing (%) in terms of different parameters in Table 2C.

## 3. Conclusions

In this research, the wound-healing ability of PDDA and PDDA/honey nanofibers was investigated for the first time. To control the release behavior of effective compounds and decrease the solubility of nanofibers, GA was used. The effect of the crosslinking duration on the different properties of nanofiber composites was also studied. We found that honey containing nanofibers exhibited an excellent antibacterial activity (99.9%) against *S. aureus* and *E. coli* bacteria. Moreover, releasing the effective compounds from nanofibers was continued up to 125 h. The prepared nanofibers were also evaluated as wound dressings for diabetic wound-healing. The results indicated that honey-containing wound dressings resulted in the best wound-healing properties mainly due to anti-inflammatory, antioxidant, and antibacterial properties of nanofibers. This process also resulted in the formation of high amounts of fibroblast cells, collagen fibers, and connective fibrils in the treated wounds.

## 4. Materials and Methods

### 4.1. Materials

Poly (diallyldimethylammonium chloride) (PDDA) with a molecular weight of Mw = 450,000 and 40 wt% in H_2_O was obtained from Sigma-Aldrich (Germany). The raw multifloral Persian Honey was obtained from a local company at Polour, Iran. Glutaraldehyde (GA) (50% V/V in water) and aluminum trichloride (AlCl_3_) were obtained from Merck (Germany). Ethanol, nutrient broth solution, phosphate-buffered saline, Folin–Ciocalteu’s phenol, Sodium carbonate (Na_2_CO_3_), methanol, and sodium citrate were of analytical grade and obtained from Merck Millipore (Germany). Sterile streptozotocin (STZ), xylazine, and ketamine were received from Sigma, St Louis, MO, USA.

### 4.2. Preparation of Nanofibers

We used the following procedure that was previously reported by our group [31]. Briefly, in order to produce the PDDA/honey nanofiber composites, we prepared two spinning solutions involving 50 and 60% *w*/*w* honey in the PDDA solution. Then, 1 mL of ethanol was mixed into the solutions under vigorous stirring to reach the volume of the mixed solution at 5 mL (room temperature for 1 h). Next, we electrospun the prepared solutions under a fixed electrical field of 20 and 17 KV for 50 and 60% *w*/*w* honey-containing solutions, respectively. We utilized the electrospinning equipment from Fanavaran nano-meghyas Co. (Iran). Final nanofiber composites were collected onto an aluminum (Al) sheet at the feeding rate of 0.8 mL/h and the distance between tip of needle and collector was set at 16 cm.

### 4.3. Crosslinking of Nanofibers

The resultant scaffolds (15 cm × 15 cm) were placed in a polyethylene-framed chamber for exposure to the vapor of 40 mL GA solution. This process was performed at different durations of 2, 3, 4, and 5 h, and the treated nanofibers were finally dried in a vacuum oven at 70 °C for 12 h to remove residual GA.

### 4.4. Assessment of Antibacterial Activity

We assessed the antibacterial properties of nanofibers against *Escherichia coli* (ATCC 25922) as Gram-negative bacteria and *S. aureus* (ATCC 25923) as Gram-positive bacteria by employing the viable cell counting method. For this purpose, 100 µL from *E. coli* and *S. aureus* suspensions were separately cultured in 100 mL of a nutrient broth solution. Next, 1 mL of the bacteria/nutrient solution was mixed into 9 mL of the sterilized nutrient broth solution (0.8%). A concentration of 5 × 10^5^ CFU/mL from testing bacteria was selected for the evaluation. The weight of the disk-shape composite samples was 100 mg with a 2.8 cm diameter. The antibacterial evaluation of nanofibers was conducted by exposing them to 10 mL of the bacteria and nutrient medium. Then, 100 µL of the solution was collected and quickly spread on a plate containing nutrient agar. Finally, plates were incubated at 37 °C for 24 h, and the surviving colonies were subsequently counted.

### 4.5. Assessment of Fluid Absorption

Fluid absorption ability (the swelling ratio) was determined via gravimetric analysis. A total of 0.1 g of nanofibers was treated in a phosphate-buffered saline (50 mL of PBS, pH = 7.4) at 35 ± 0.1 °C for 24 h in order to obtain the maximum swelling equilibrium, which was then calculated as Equation (1).

(1) $$Q=\frac{m_{a}-{\;m}_{b}}{m_{b}}$$

where m_b_ (g) is the weight of scaffolds in the dry state and m_a_ (g) is the weight of the scaffolds after swelling at a specific time period, respectively [54].

### 4.6. Assessment of Solubility of Nanofibers

Nanofibers were evaluated after immersion in a distilled water bath for 360 min (25 °C and pH = 7.4). In the next step, the treated materials were dried at 60 °C for 6 h. The solubility of samples was assessed using the following equation:

(2) $$\mathrm{Solubility}\;\left(\%\right)=\frac{m_{X}-{\;m}_{y}}{m_{y}\;}\times 100$$

where m_x_ and m_y_ are the weight of nanofibers before and after immersion in distilled water, respectively.

### 4.7. Evaluation of Phenolic and Flavonoid Compounds

The amount of total phenolic compounds was measured using Folin–Ciocalteu reagent [43]. Briefly, 7.5 mL of distilled water was mixed with 0.3 mL of Folin–Ciocalteu’s phenol (diluted 1:10). Then, 1 mg of the nanofiber sample was dipped in the resultant solution for 3 min. Next, 1 mL of Na_2_CO_3_ in deionized water (20% *w*/*v*) was added to the mixture. The sample was left in this solution for 1 h at room temperature and in a dark environment, and the absorbance was then assessed at 760 nm using a single beam UV spectrophotometer. Final evaluation was conducted as gallic acid equivalents (mg of gallic acid (GAE)/mg dry weight honey).

The total flavonoid compounds were measured using the Dowd method described elsewhere [55]. Briefly, 5 mL of 2% aluminum trichloride (AlCl_3_) in methanol was mixed with the same volume of a honey solution (0.01 mg/mL). After 10 min, absorption was measured at 415 nm against a blank sample containing of a 5 mL honey solution with 5 mL methanol without AlCl_3_. The total flavonoid content was determined using a standard curve with quercetin (0–50 mg/L) for comparison. Total flavonoid content was expressed as mg of quercetin equivalents (QE)/100 g of honey.

### 4.8. Evaluation of Total Antioxidant Capacity

The antioxidant activities were evaluated as reported by researchers and expressed relative to ascorbic acid [55]. In this test, methanol was used as a blank sample. The reaction material was mixed in a vortex mixer and left in a water bath at 95 °C for 90 min. Absorbance values were measured at the wavelength of 695 nm and the antioxidant activity was calculated as ascorbic acid equivalents (mg AAE/1 g honey).

### 4.9. Conducting Diabetic and Wound-Healing Tests

Male Wistar rats with an average weight of 150–200 g were taken for diabetic studies. Animals were first kept at room temperature (23 °C) in a 12 h light/dark cycle, with access to standard rodent chow and water. Testing was carried out according to Islamic Azad University (Rodehen Branch) ethical guidelines, and the ethical permission code was IR.IAU. R.REC.1400.006.

Diabetic tests were conducted in rats by a single intraperitoneal injection of 70 mg/kg body weight of sterile streptozotocin (STZ) (Sigma, St Louis, MO, USA) in sodium citrate (0.1 mol/L, pH 4.5). Diabetic rats with a blood glucose level of 300 mg/dL or higher were selected. In the next step, they were anesthetized via the intraperitoneal injection of xylazine (13 mg/kg) and ketamine (66.7 mg/kg). We shaved the dorsal hair of rats, and 1.5 cm diameter full thickness wounds were generated with a biopsy punch. Then, three groups of rats, each containing five rats, were randomly chosen. The wounds of the animals were exposed to PDDA/honey (40/60) and PDDA/honey (50/50) nanofibers, and the groups were classified as group 1 and 2, respectively. The conventional gauze bandage and PDDA nanofiber composite-treated wounds were considered as controls.

The wound-healing of rats was assessed through captured photographs at different time periods. The non-healed spots were measured by a computer analytical system. The healing rate was also evaluated with the following equation:

(3) $$\mathrm{Healing}\;\mathrm{rate}\;\left(100\%\right)=\frac{\mathrm{primitive}\;\mathrm{area}-\mathrm{nonhealing}\;\mathrm{area}}{\mathrm{primitive}\;\mathrm{area}}\times 100$$

We also monitored the wounds of those rats for 3 days (*n* = 5), 7 days (*n* = 5), and 14 days (*n* = 5) after treatment.

### 4.10. Histopathological Analysis

Formalin-fixed paraffin-embedded wound tissue samples were cut into 4 μm tissue sections. Then, they were stained with hematoxylin–eosin, further evaluated under a light microscope, and graded with respect to epidermal regeneration, granulation tissue formation, and the angiogenesis and migration of fibroblast cells.

### 4.11. Histomorphometry Analysis

On the 14th day, epithelialization was semi-quantitatively evaluated by using the following scale: 0 (without new epithelialization), 1 (25%), 2 (50%), 3 (75%), and 4 (100%). Sections were also semi-qualitatively considered to be related to angiogenesis. Then, they were assessed according to the number of new vessels within the scar tissue, according to the following scale: 0 (none), 1 (few), 2 (moderate), 3 (many), or 4 (considerably). In these evaluations, results were reviewed by an independent observer blinded to the treatment groups. In our histomorphometric evaluation, neovascularization and collagen density were also monitored and evaluated by using Image-Pro Plus^®^ V.6 software (Media Cybernetics, Inc., Rockville, MD, USA).

### 4.12. Design of Experiments

In this study, we utilized experimental design software (Design Expert Software V 11.0.3) and Response Surface Methodology (RSM) with Central Composite design, and results are presented in Table 2A. Some versions were routinely duplicated to create the software reliability which could minimize errors.

According to Table 2A, the lowest wound-healing rates were those of Experiment 24, which involved 12 h of wound dressing. However, the highest percentage of improvement was observed in the Experiment 30, which was due to the combination of the desired percentage and the appropriate duration of use. In general, according to the results, it can be concluded that the incorporation of honey in wound dressing had a significant effect and led to improved wound-healing properties.

Due to the fact that the test validity of the RSM model was less than 0.05 (in the amount of 0.0001), we found that different experimental variables had significant influence on wound-healing (Table 2B). According to F-statistics, the influence of different variable parameters on wound dressing can be displayed in the following order: wound dressing days, PDDA (%), and honey (%). Therefore, wound dressing days are revealed to have the most effect on wound-healing properties. Furthermore, P-statistics reached less than 0.05% for all variables except the duration of treatment with GA (h). These results from the ANOVA test also illustrated that this parameter did not have a significant effect on wound-healing; however, a significant change was observed in the quadratic equations of the GA executive model. We should further highlight that the effect of the different variables can be observed by using the three-dimensional diagram as well as the executive model, and the mathematical model of the designed experiment can be extracted by using Equation (4):

Wound-Healing: 70.4104 + −9.05634 × [honey] + 2.4282 × [PDDA] + 0.23608 × [GA] + 34.6683 × D + 15.5353 × [honey] [PDDA] + −22.2139 × [honey] [GA] + 5.96673 × [honey] [Day] + −16.7113 × [PDDA] [GA] + 10.4691 × [PDDA] [Day] + −5.06506 × [GA] [Day] + 23.3817 × [honey] 2 + −3.49 × [PDDA] 2 + −9.04543 × [GA] 2 + −14.9567 × [Day] 2 + −7.54687 × [honey] [PDDA] [GA] + 6.34389 × [honey] [PDDA] [Day] + −10.2752 × [honey] [GA] [Day] + −16.9153 × [PDDA] [GA] [Day] + 5.19699 × [honey] 2 [PDDA] + −0.805398 × [honey] 2 [GA] + 0 × [honey] 2 [Day] + 0 × [honey] [PDDA] 2 + 20.1858 × [honey] [GA] 2 + 2.4856 × [honey] [Day] 2 + 0 × [PDDA] 2 [GA] + 0 × [PDDA] 2 [Day] + 0 × [PDDA] [GA] 2 + 0 × [PDDA] [Day] 2 + 0 × [GA] 2 [Day] + 0 × [GA] [Day] 2 + 0 × [honey] 3 + 0 × [PDDA] 3 + 0 × [GA] 3 + −9.02667 × [Day] 3(4)

**Figure 9:**
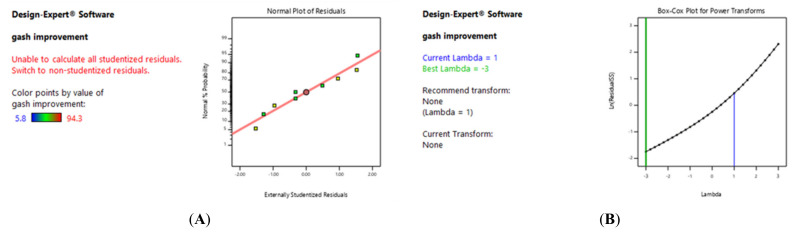
(**A**) Dispersion diagram of normal test distribution; (**B**) box Cox diagram of the healing rate.

Figure 9A showed the scatter plot for the normal distribution of the conducted experiments. Despite the availability of regular data, some median distributions were expected. We should also mention that specific curved patterns, such as “S-shapes”, were easily recognizable. Therefore, a better analysis could be achieved by performing the transfer function on the dependent variable or model response. In this study, the data followed the central normal line and all experiments were statistically normal and acceptable. A box Cox diagram (Figure 9B) was utilized as a tool to identify the most appropriate power transfer function that could be applied to the response. The lowest point in the box Cox diagram indicated the best Landa value at which the least squares in the converted model were generated. When the ratio of maximum to minimum response value was greater than three, we observed a greater ability to improve the model using the power function. According to the diagram for the difference between a minimum and a maximum of 3, we did not further consider a power improvement for the experimental model. Our chart also demonstrated that the 95% confidence interval was achieved and Landa simulated the creation of a mathematical computational link between variables.

## Figures and Tables

**Table 1:** Histomorphometric analysis of wounds after treatment with different dressings.

Sample	Angiogenesis	Epitheliogenesis (Score)	Inflammatory Cells/3HPF
PDDA/honey nanofiber composite (40/60)	0 (3 days) 2 (7 days) 3 (14 days)	0 (3 days) 2 (7 days) 4 (14 days)	179 (3 days) 85 (7 days) 24 (14 days)
PDDA/honey nanofiber composite (50/50)	0 (3 days) 2 (7 days) 2 (14 days)	0 (3 days) 1 (7 days) 2 (14 days)	161 (3 days) 121 (7 days) 80 (14 days)
PDDA nanofiber	0 (3 days) 1 (7 days) 2 (14 days)	0 (3 days) 0 (7 days) 1 (14 days)	98 (3 days) 132 (7 days) 1 (14 days)
Gauze bandage	0 (3 days) 0 (7 days) 1 (14 days)	0 (3 days) 0 (7 days) 0 (14 days)	40 (3 days) 112 (7 days) 99 (14 days)

**Table 2:** (**A**) An experimental design used to evaluate wound-healing properties (%), (**B**) ANOVA analysis (%) used in this study, and (**C**) optimum values for wound-healing (%) regarding the different parameters used in this study.

(**A**)					
**Run**	**A:** **Honey (%)**	**B:** **PDDA** **(%)**	**C:** **Duration of GA Treatment** **(h)**	**D: Day**	**Response: Wound-Healing (%)**
1	60	60	5	14	72.2
2	60	40	5	14	74.6
3	50	50	6.5	7	18
4	40	60	5	14	69.7
5	60	40	2	14	68
6	60	60	5	3	33.3
7	50	50	3	14	81.7
8	40	60	3	7	59.8
9	40	60	3	3	21.8
10	40	60	5	3	35
11	40	60	2	14	67.8
12	50	70	3	7	30.2
13	50	50	3	18.5	10
14	60	60	2	3	34.2
15	60	40	2	14	69
16	50	50	3	7	46.7
17	60	40	5	3	35.2
18	60	40	2	3	36.8
19	40	40	5	3	32.2
20	50	50	3	7	45.9
21	70	30	3	7	21.2
22	50	30	3	7	34
23	50	50	3	7	45.5
24	50	50	3	0.5	9
25	40	60	5	14	69
26	50	50	3	7	45.9
27	50	50	3	7	46.3
28	40	40	2	3	34.5
29	50	50	0.5	7	5.8
30	40	60	3	14	94.3
(**B**) (ANOVA with Cubic Model (Aliased))					
**Source**	**Sum of Squares**	**df**	**Mean Square**	**F-Value**	***p*-Value**
Model	15450.29	23	671.75	2555.81	<0.0001
A-honey	14.87	1	14.87	56.56	0.0003
B-PDDA	18.50	1	18.50	70.40	0.0002
C-GA	0.3446	1	0.3446	1.31	0.2958
D-Day	1388.99	1	1388.99	5284.67	<0.0001
AB	265.60	1	265.60	1010.51	<0.0001
AC	292.99	1	292.99	1114.75	<0.0001
AD	82.14	1	82.14	312.53	<0.0001
BC	138.97	1	138.97	528.76	<0.0001
BD	205.19	1	205.19	780.70	<0.0001
CD	215.62	1	215.62	820.38	<0.0001
A^2^	842.77	1	842.77	3206.47	<0.0001
B^2^	278.40	1	278.40	1059.24	<0.0001
C^2^	1733.32	1	1733.32	6594.75	<0.0001
D^2^	3534.93	1	3534.93	13449.31	<0.0001
ABC	262.71	1	262.71	999.54	<0.0001
ABD	82.43	1	82.43	313.64	<0.0001
ACD	106.69	1	106.69	405.94	<0.0001
BCD	342.15	1	342.15	1301.77	<0.0001
A^2^B	40.44	1	40.44	153.88	<0.0001
A^2^C	2.84	1	2.84	10.80	0.0167
A^2^D	0.0000	0	-	-	-
AB^2^	0.0000	0	-	-	-
AC^2^	209.43	1	209.43	796.80	<0.0001
AD^2^	7.02	1	7.02	26.72	0.0021
B^2^C	0.0000	0	-	-	-
B^2^D	0.0000	0	-	-	-
BC^2^	0.0000	0	-	-	-
BD^2^	0.0000	0	-	-	-
C^2^D	0.0000	0	-	-	-
CD^2^	0.0000	0	-	-	-
A^3^	0.0000	0	-	-	-
B^3^	0.0000	0	-	-	-
C^3^	0.0000	0	-	-	-
D^3^	1255.82	1	1255.82	4778.02	<0.0001
Pure Error	1.58	6	0.2628	-	-
Cor Total	15,451.87	29	-	-	-
(**C**)					
**Honey (%)**	**PDDA (%)**	**Duration of GA Treatment (h)**	**DAY**	**Wound-Healing (%)**	**Desirability**
51.5	48.5	2.571	12	100	1.000
41	59	3.5	12	98	1.000
60	40	2	14	100	1.000

## Data Availability

All data generated or analyzed during this study are included in this published article.
